# Research on the Weld Position Detection Method for Sandwich Structures from Face-Panel Side Based on Backscattered X-ray

**DOI:** 10.3390/s19143198

**Published:** 2019-07-20

**Authors:** Angang Wei, Baohua Chang, Boce Xue, Guodong Peng, Dong Du, Zandong Han

**Affiliations:** 1 Department of Mechanical Engineering, Tsinghua University, Beijing 100084, China; 2 Key Laboratory for Advanced Materials Processing Technology, Ministry of Education, Beijing 100084, China

**Keywords:** web-core sandwich panels, aluminum T-joint, T-joint from face-panel side, weld position detection, X-ray backscatter, denoising method

## Abstract

Web-core sandwich panels are a typical lightweight structure utilized in a variety of fields, such as naval, aviation, aerospace, etc. Welding is considered as an effective process to join the face panel to the core panel from the face panel side. However, it is difficult to locate the joint position (i.e., the position of core panel) due to the shielding of the face panel. This paper studies a weld position detection method based on X-ray from the face panel side for aluminum web-core sandwich panels used in aviation and naval structures. First, an experimental system was designed for weld position detection, able to quickly acquire the X-ray intensity signal backscattered by the specimen. An effective signal processing method was developed to accurately extract the characteristic value of X-ray intensity signals representing the center of the joint. Secondly, an analytical model was established to calculate and optimize the detection parameters required for detection of the weld position of a given specimen by analyzing the relationship between the backscattered X-ray intensity signal detected by the detector and the parameters of the detection system and specimen during the detection process. Finally, several experiments were carried out on a 6061 aluminum alloy specimen with a thickness of 3 mm. The experimental results demonstrate that the maximum absolute error of the detection was 0.340 mm, which is sufficiently accurate for locating the position of the joint. This paper aims to provide the technical basis for the automatic tracking of weld joints from the face panel side, required for the high-reliability manufacturing of curved sandwich structures.

## 1. Introduction

**Figure 1:**
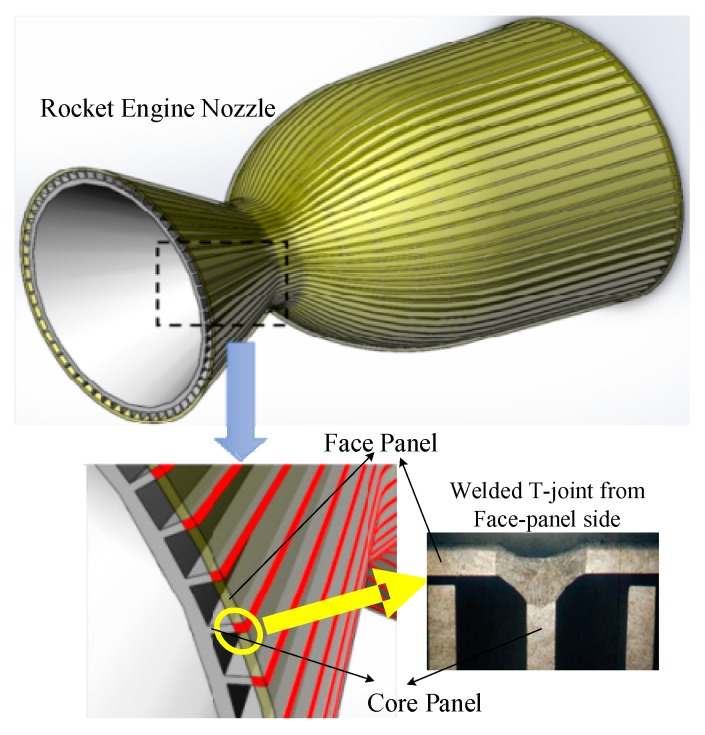
Welded T-joint from the face panel side in the sandwich structure for a rocket engine nozzle.

Web-core sandwich panels are lightweight structures with high specific strength and high specific stiffness. The structure is utilized in a variety of fields, such as aviation, aerospace, the marine shipbuilding industry, etc. [1,2,3,4]. The structure consists of two face panels and a periodic core made from webs (Figure 1). Welding is considered as an effective process to join the face panel to the core panel through a T-joint. Considering the structural characteristics of the sandwich panels, it is sometimes impractical to weld such T-joints using conventional fillet welds, which is implemented from the core panel side. Instead, the welding must be implemented from the face panel side (backside of the fillet T-joint) to join the face panel to the core panels [5,6]. Many researchers have conducted in-depth studies of the influence of welding from the face panel side on the sandwich structure. The fundamental mechanical properties of hull steel CCS-B sandwich plate laser-welded joint and base metals were presented in [7]. Li et al. [8] built a mathematical model of the laser welding of steel with a T-joint to investigate the formation process of keyhole-induced porosity. Romanoff et al. [9] proposed a novel approach to the structural analysis of patch-loaded laser-welded web-core sandwich panels which are sensitive to the response of the T-joint. Authors in [4,10] built a prediction model of weld formation for the laser-welded T-joint from the face panel side. Yong [11] studied the effect of the laser process on the dissimilar-metals welding of AZ31B magnesium alloy and 304 stainless steel in the sandwich structure. In contrast to conventional joint welding, this special welding mode for the T-joint from the face panel side of the sandwich structure presents new challenges. To guarantee the welding quality, it is necessary to implement weld position detection—especially when the welding heat source is not aligned to the core panel due to assembly errors or thermal deformation.

As far as welded T-joints from the face panel side are concerned, weld position detection is pivotal for weld formation and joint performance. When the deviation of panel position becomes out of range, it leads to poor formation and unqualified joints. High-accuracy positioning of the welding may create good-quality welds with high precision and repeatability. Thus, it is necessary to detect the position of the core panel under the face panel when welding the T-joint from the face panel side. However, the face panels shield the core panels during the welding of the T-joint, which leads to “blind welding”. Some conventional weld position detection methods are not applicable in this special welding mode for T-joints. Accordingly, some researchers have conducted explorations of special detection technologies for T-joint welding from the face panel side. A special detection method for friction-stir-welded T-joints based on axial force has been reported in some works [12,13], where the deviation state from the center of the weld seam can be judged by detecting the feedback of the specimen’s axial force to the stir head. A method for detecting the welding deviation state by observing an image of the plasma vapor while the molten pool overflows the specimen during the welding process was proposed in [14]. Another detection method based on backscattered X-rays has also been reported, and a preliminary study was carried out on a nickel-based alloy specimen with thickness 0.8 mm [15,16]. So far, the detection method based on axial force is only targeted at the weld position detection of friction-stir-welded T-joints, and is not available in other welding processes. Deviation detection based on plume images may fail in conditions where the molten pool remains inside the specimen and there are molten droplets or downward plumes, and it is difficult for large sandwich structures to capture the upward and downward plume images simultaneously during the welding process. Few investigations have been reported in the literature on weld position detection for T-joints in web-core sandwich structures using X-ray-based methods, where safety issues in the X-ray environment and the applicability to variable conditions (e.g., the dynamic detection performance under high welding speeds) are considered as the major problems that restrict the further development and application of the X-ray method. Accordingly, this work made efforts to reduce the radiation dose and improve the applicability of the X-ray method under variable conditions. Moreover, lighter materials (e.g., aluminum) and thicker panels which were not involved in the existing research have gained in popularity for sandwich structures in various fields, especially aviation and shipbuilding. Therefore, aluminum web-core sandwich panels were chosen to study the weld position detection of T-joints from the face panel side, aiming to provide a technical basis for the manufacturing of such sandwich structures. First, this paper analyzes the characteristics of the backscattered X-ray intensity signal and investigates an appropriate processing algorithm to extract the characteristic value of the acquired X-ray intensity signals representing the center of the T-joint. Then, this paper analyzes the relationship between the detected backscattered X-ray intensity signal and the parameters of the detection system and specimen, and develops an analytical model to calculate and optimize the detection parameters required for detecting the joint position of a given specimen. Finally, several experiments were carried out on an AA6061 aluminum alloy specimen with a thickness of 3 mm; the influence of signal processing algorithms and sampling frequency on the detection results were investigated in terms of detection accuracy and dynamic performance. 

## 2. Detection Method and Characteristics of X-ray Intensity Signal

**Figure 2:**
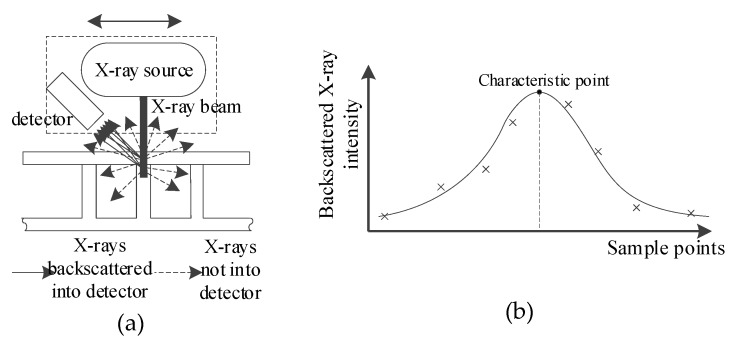
The scheme for X-ray scanning to locate the center of core panel: (**a**) the scheme for scanning; (**b**) the scheme to locate the core panel.

Weld position detection based on backscattered X-rays utilizes a collimated X-ray beam to scan the specimen along the cross section of a weld, as shown in Figure 2. The intensity of the backscattered X-ray is acquired at different scanning positions, and then processed utilizing curve fitting to obtain the reference point which represents the center position of the T-joint (i.e., the center position of the core panel).

The key to detecting the T-joint position based on backscattered X-rays lies in locating the core panel under the face panel. While the well-collimated pencil-beam of the X-ray is irradiated at and penetrated through the face panel, the cumulative intensity of backscattering X-rays increases with the increase of the penetrated specimen volume. When the X-ray beam irradiates at the center of the core panel, the irradiated volume reaches the maximum and the backscattered X-ray intensity at this time is also a maximum value. Meanwhile, to reduce the attenuation length of X-ray and facilitate the adjustment of the relative position between X-ray source, detector, and specimen during the detection process, the X-ray source is utilized perpendicular to the face panel of specimen.

X-ray intensity is expressed by the counted value of photons in unit time [17]. The counted value is a random variable, which obeys a Poisson distribution [18]. Assuming that the average number of X-ray photons measured by the detector per unit time is $\bar{N}$, the probability that the number of X-rays actually detected by the detector per unit time falls within the interval $\left[\bar{N}-\sqrt{\bar{N}},\bar{N}+\sqrt{\bar{N}}\right]$ is 68.3%. The degree of scatter of the measured values is expressed by the relative standard deviation, and the standard deviation of the measured values can be estimated as follows:

(1) $$\sigma \;\approx \;\sqrt{\bar{N}}\;\approx \;\sqrt{N}.$$

The relative standard deviation of the measured values can also be obtained by:

(2) $$\nu =\frac{\sigma }{N}\approx \frac{\sqrt{N}}{N}=\frac{1}{\sqrt{N}}.$$

**Figure 3:**
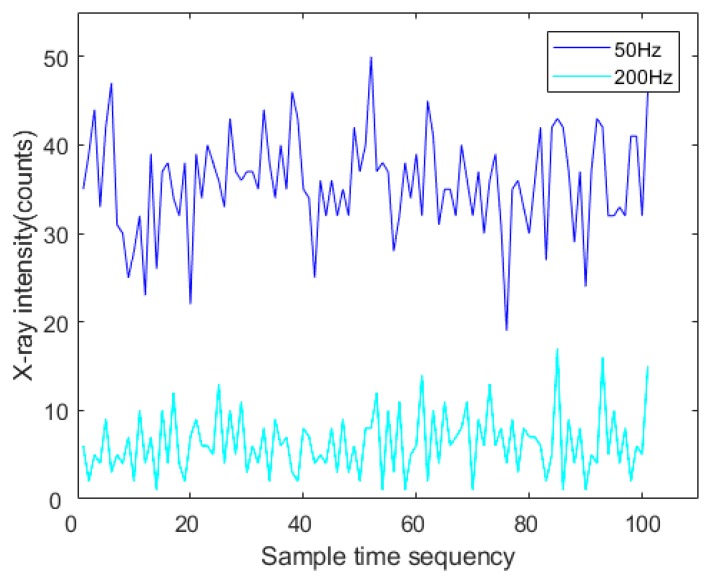
The original signals at different sampling frequencies in the same specimen position.

As can be seen from the above analysis, the actual measured value has a large fluctuation, which can be regarded as the interference of noise on the accurate signal. The blue curve in Figure 3 is the detected X-ray intensity signals acquired for the same non-weld location at different times. From Figure 3, it can be seen that the actual intensity values acquired for the same position were not the same even when the whole set of detection parameters remained constant. Therefore, it was necessary to process the original signal through an appropriate denoising algorithm to more accurately extract the characteristic value of the X-ray intensity signals representing the center position of the core panels.

**Figure 4:**
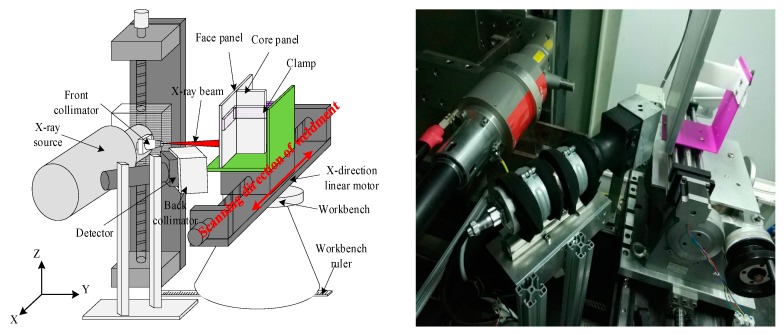
Schematic diagram (**left**) and the real experimental system (**right**).

On the other hand, the acquired X-ray intensity signal is the counted value of X-ray photons measured by the detector in a single sampling period, so the selection of the sampling frequency also has an influence on the X-ray intensity signal. Figure 4 shows the acquired backscattered X-ray intensity signals at a non-weld position with two different sampling frequencies at a certain X-ray source intensity. As shown in Figure 4, when the sampling frequency increased, the acquired X-ray intensity signal decreased, and the relative standard deviation of the signal increased according to Equation (2). While the sampling interval (sampling accuracy) remained constant, the higher sampling frequency led to a shorter time to finish one scan of the weld cross section. Thus, the higher sampling frequency was able to support the faster welding speed. Under the premise of ensuring the sampling accuracy, a relatively lower sampling frequency can be appropriately selected to reduce the dispersion degree of the acquired X-ray intensity signals.

## 3. Experimental System for Weld Detection

In order to verify the validity of the proposed detection method, an X-ray backscattering detection system was designed. The schematic diagram of the experimental apparatus is shown in Figure 4. It consisted of five parts: the X-ray source, the front collimator, the back collimator, the X-ray intensity detection system, and the X-direction linear motor. The X-ray source used was a Comet MXR-225, which is capable of emitting X-ray beams with energy up to 225 keV. The front collimator was used to collimate the beam emitted by the X-ray source. The back collimator was used to shield the X-ray photons scattered from other directions except for that irradiated by the incident X-ray beam and reduce the noise of the acquired X-ray intensity signal. The X-ray intensity detection system was used to acquire and transmit the X-ray intensity signal at a certain sampling frequency. The T-joint specimen to be detected was moved by the X-direction linear motor in the direction perpendicular to the incident X-ray beam. The center line of the detector, the X-ray source, and the front collimator were located in the same horizontal plane. The scattering angle between the center line of the detector and the incident beam was 120°. During the actual detection process, the X-ray source was moved back and forth to complete the scanning of the specimen. In this paper, in order to simplify the experimental system, the T-joint moved linearly while the X-ray source was fixed.

**Figure 5:**
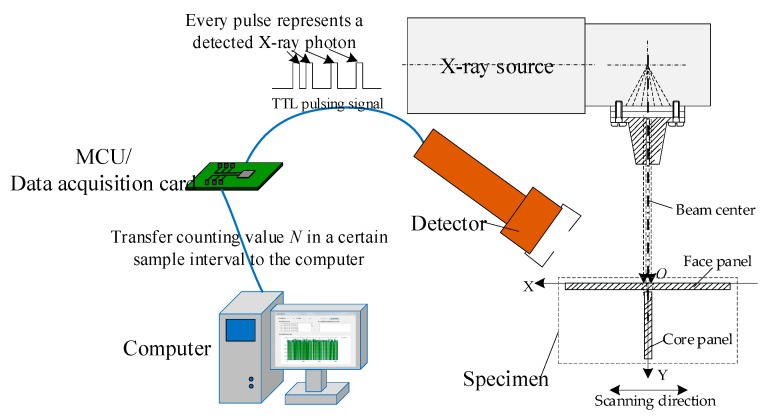
Schematic diagram of backscattered X-ray intensity signal acquisition. MCU: micro control unit.

A schematic diagram of the backscattered X-ray intensity signal acquisition system is shown in Figure 5. The upper surface of the face panel was set as the *X*-axis, the intersection point between the center line of incident beam and the *X*-axis was set as origin O. The X-ray intensity signal acquisition system consisted of three parts: a signal detector, an MCU (micro control unit), and a computer. The detector consisted of a scintillator and a photomultiplier tube, which generated a pulse signal when an X-ray photon was detected, then the MCU counted the number of the pulse signals in a certain sample interval and transmitted the counted values to the computer as acquired X-ray intensity signals. Finally, the computer processed the signal and also acted as the human–machine interface of the experimental system.

## 4. Acquisition and Processing of the Backscattered X-ray Intensity Signal

### 4.1. Signal Acquisition

**Figure 6:**
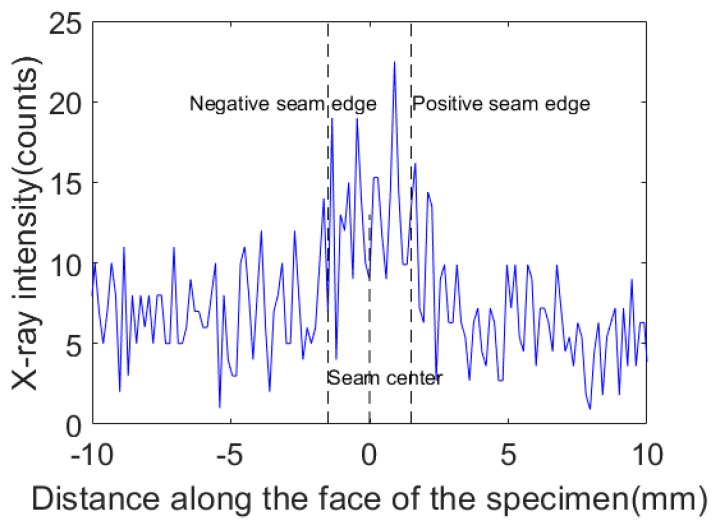
The X-ray intensity signals at different weld positions in a single scanning period.

The specimen consisted of a face panel and a core panel, forming a T-joint as shown in Figure 5. The face panel was made of a 3-mm-thick 6061 aluminum alloy that was 300 mm long and 150 mm wide, while the core panel was made of 3-mm-thick 6061 aluminum alloy with 300 mm length and 50 mm width. As described in Section 2, a higher sampling frequency was selected while ensuring the signal-to-noise ratio (the signal was the difference between intensities at the weld center and the non-weld position, and the noise was the signal noise at the weld center). Figure 6 shows the X-ray intensity signals acquired by completing a single scan of the weld. The sampling frequency was 200 Hz. For a weld with 3 mm width, 20 sampling points were acquired across the cross section of the weld with a sample distance interval of 0.15 mm.

As shown in Figure 6, the acquired X-ray intensity signals contained nonstationary random disturbance, and the detected signal was not completely symmetrical with respect to the center of the weld. Furthermore, the abrupt changes in signal intensity were not detected at the seam edges because the X-ray beam was not focused very well. The diameter of the spot became larger as the collimated beam reached the upper face of the specimen. The original diameter of the spot was 2 mm when emitted from the collimator, but it became larger than the thickness of core panel when reaching the upper face of the specimen. Therefore, even at a non-weld position within a certain distance from the seam edge, the core panel was still under the illumination of the X-ray beam, and the intensity of backscattered X-ray did not decrease sharply. 

### 4.2. Signal Processing

#### 4.2.1. Denoising

**Figure 7:**
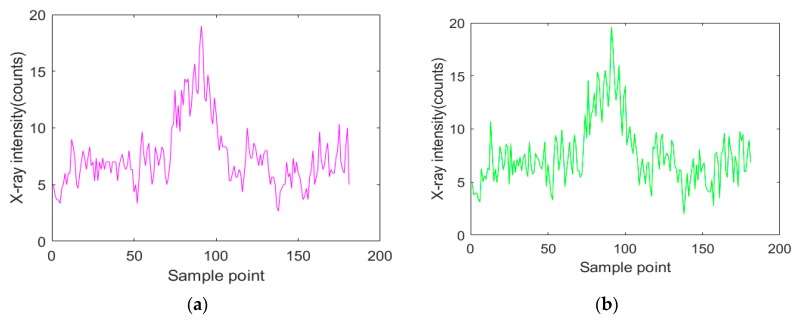

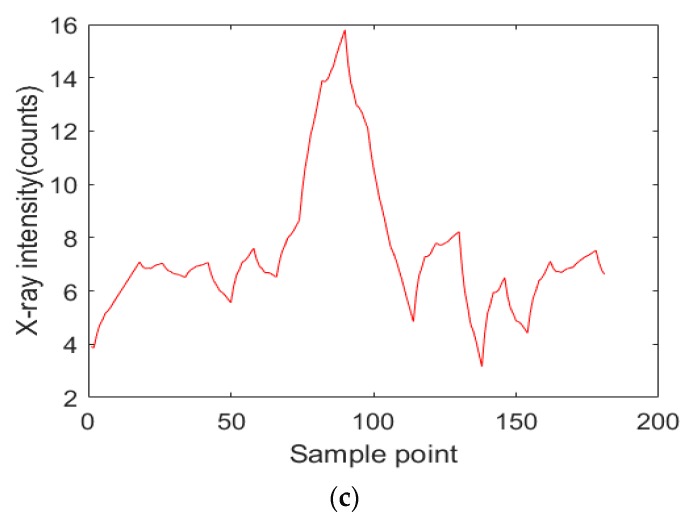
The signal processing results using different denoising algorithms: (**a**) the average filter; (**b**) the Kalman filter; (**c**) the wavelet packet.

In the actual detection, the acquired X-ray intensity signals contained nonstationary random disturbance because of the characteristics of X-rays (Figure 7). In order to extract the characteristic values representing the weld center position in the acquired signals, several signal processing methods were studied. According to different purposes and signal characteristics, several denoising algorithms were introduced. For signals containing nonstationary random noise (e.g., the X-ray intensity signal acquired in this paper), the average filter method is available for eliminating the nonstationary random noise [19,20]. Suppose *S_k_* is the original signal at the sample point *k*, the output value $S_{k}^{\prime }$ after the application of the average filter would be:

(3) $$S_{k}^{\prime }=\frac{1}{l}\sum_{j=k-l+2}^{k+l-2}S_{k+j},$$

where *l* is the length of the average filter. In order to maintain the abrupt waveform of the original signal, a shorter mean filter length of three was used in this paper, but the average filter method may lose signal details and suppress the maximum value of the signals. It reduced the signal-to-noise ratio of the signal and was not conducive to the extraction of the characteristic values of signals, as shown in Figure 7a. The Kalman filter method can also be used to effectively eliminate the random noise [21,22]. Suppose the original signal is *S*(*n*), *n* ∈ [1,*M*], *S_k_* is the original signal at sample point *k*, the state equation and measurement equation are given by:

X_k_ = A × X_k − 1_ + Q_k_, (4)

*S_k_* = H × X_k_ + R_k_, (5)

where X_k_ and X_k − 1_ are the exact state values at sample points k and k − 1, A is the state transition matrix, H is the measurement matrix of detector, and A and H are taken as unit values. Q_k_ is the state noise, taken as 1. The previous updated state value was taken as the present prediction state. R_k_ is the measurement noise of detector at sample point k. As described in Section 2, the measurement values mainly fell within the interval of $\left[\bar{N}-\sqrt{\bar{N}},\bar{N}+\sqrt{\bar{N}}\right]$, and R_k_ was taken as $\sqrt{S_{k}}/\;$2.

The prediction equation at sample point k is:

X(k|k − 1) = A × X(k − 1|k − 1),(6)

where X(k|k − 1) is the prediction state value at sample point k based on the updated state value at sample point k − 1, X(k − 1|k − 1) is the updated state value at sample point k − 1.

The variance of prediction is obtained by:

P(k|k − 1) = A × P(k − 1|k − 1) × A^T^ + Q_k_, (7)

where P(k|k − 1) is the variance of prediction state X(k|k − 1). P(k − 1|k − 1) is the variance of the updated state X(k − 1|k − 1).

The Kalman gain is obtained by the variance of prediction state:

K(k) = [P(k|k − 1) × H^T^]/[H × P(k|k − 1) × H^T^ + R_k_]. (8)

The updated state is obtained by:

X(k|k) = X(k|k − 1)+K(k) × [S_k_ − H × X(k|k − 1)], (9)

where X(k|k) is the updated state at sample point k and the output signal after Kalman filtering.

The updated variance is obtained by:

(10) P(k|k) = P(k|k−1)−K(k)×H×P(k|k−1),

where X(k|k) and P(k|k) are utilized to obtain the updated state at sample point k + 1. The acquired signals are processed as Equations (4)–(10). This method not only suppressed the maximum value but also made the signal waveform slightly deformed, as shown in Figure 7b. The wavelet packet analysis method can provide better decomposition for a given signal, so it performs better in local analysis in time and frequency domains. In addition, wavelet packet analysis is suitable for obtaining the signal mutation. The waveform of the processed signal is also retained well [23,24,25]. Suppose the original signal is *S*(*n*), *n* ∈ [1,*M*]. The data queue *S*(*P*) with length *P* from *S*(*n*) is selected and then wavelet packet decomposition is run for three layers. The best threshold is sought to quantize the coefficients of wavelet packets. Finally, the denoised X-ray intensity signal *S*’(*P*) can be obtained by reconstructing with the best basis of wavelet packets. Therefore, the wavelet packet method is suitable for processing signals with nonstationary random noise and mutation signals. From Figure 7c, we can see that the non-stationary random noise originally contained in the original X-ray intensity signal was significantly eliminated by the wavelet-packet-based denoising algorithm.

On the other hand, as shown in Figure 5, the detector was located on the positive side in the X-direction. While the specimen moved to symmetrical positions on both sides of the origin O, the position on the positive side of the X-direction was closer to the detector. The detected X-ray intensity signal at the positive side was greater than the signal at the negative side. So, the detected signal was not completely symmetrical about the origin O. Asymmetrical signals lead to signal waveform offset and larger detection error. To overcome the influence of the detector asymmetry on the accuracy of the derived characteristic values as well as the detection errors, the denoised signals have to be correct before fitting.

According to the above analysis, it can be seen that there was nonstationary random noise contained in the acquired X-ray signals. At the same time, the acquired X-ray signals were not strictly symmetrical about the center of the weld due to the asymmetry of the detector’s location relative to the specimen. Based on the analysis of different processing algorithms, a processing algorithm based on wavelet packet was designed. The algorithm procedure is as follows:

1. Suppose the original signal is *S(n)*, *n* ∈ [1,*M*], select a data queue *S(P)* with a length of *P* from *S(n)*, where *P* is the number of sampling points acquired in single scanning period, *S(P)* = {*S_1_, S_2_, S_3_*, *…*, *S_P_*}.
2. Read into the select sampling signal *S(P)*, then do wavelet packet decomposition for L layers.
3. Look for the best threshold to quantize the coefficients of wavelet packets.
4. Reconstruct the signal with the best basis of wavelet packets, and then get the denoised X-ray intensity signal *S’(P).*
5. Compare all data of *S’(P)* to find the maximum *S_i_’*
  *S_i_*’ = max{*S_1_*’, *S_2_*’, *S_3_*’, …, *S_P_*’}.
6. Correct the denoised signal *S’(P)*.
  While scanning in the positive X-direction, the sample signals acquired after the maximum sampling signal (*S_i_’)* are multiplied by the correction coefficient.
  *S*”(*P*) = {*S_1_*’, *S_2_*’, *S_3_*’, …, *S_i − 1_*’, *S_i_*’, *S_i + 1_*’ × α, …, *S_P_*’ × α}
  The coefficient is obtained by minimizing the sum of the differences of symmetric signals about the maximum value.
  $$\forall .\alpha \;\min\;\sum_{n=1}^{i-1}\left(S_{i+n}^{\prime }\times \alpha -S_{i+n}^{\prime }\right)$$
  While scanning in the negative X-direction, the sample signals acquired before the maximum sampling signal (*S_i_’)* are multiplied by the correction coefficient.
  *S*”(*P*) = {*S_1_*’ × α, *S_2_*’ × α, *S_3_*’ × α, …, *S_i − 1_*’ × α, *S_i_*’, *S_i + 1_*’, …, *S_P_*^’^}

**Figure 8:**
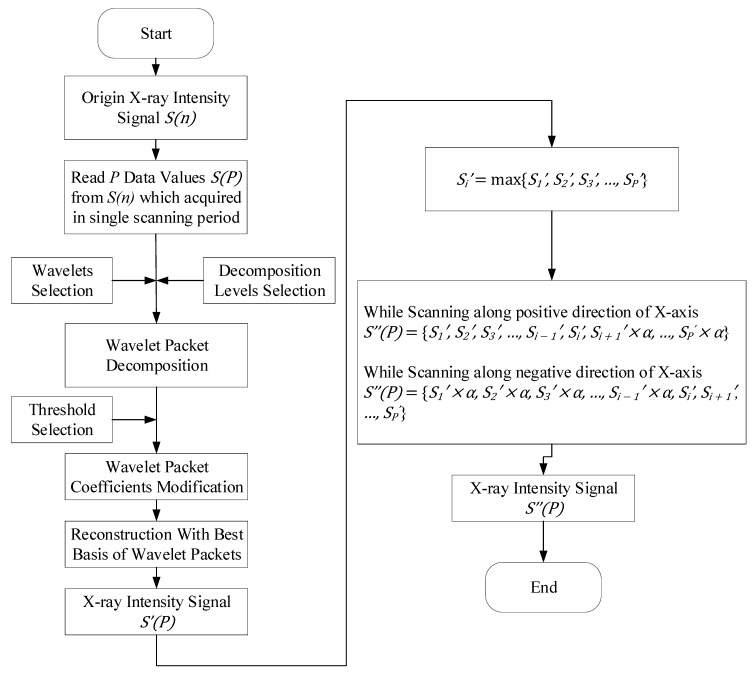
The flowchart of processing algorithms.

Figure 8 shows a flowchart of the processing algorithms for the X-ray intensity signals. The nonstationary random noise contained in the original X-ray intensity signals was eliminated effectively by using the wavelet-packet-based processing algorithm (see Figure 10d).

#### 4.2.2. Curve Fitting

**Figure 9:**
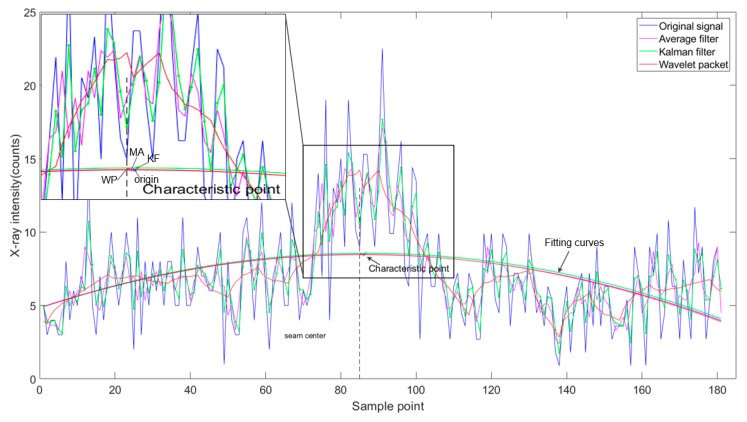
The curve fitting of some denoising algorithms.

After denoising and correction, curve fitting was performed on the denoised X-ray intensity signals by using a least squares method, and then the characteristic point representing the center position of the weld in the fitted curve was determined, as shown in Figure 9. The characteristic point was the maximum value of the fitting curve of the denoised X-ray intensity signals. The absolute error of detection was determined by comparing the derived weld position with the real calibrated seam center.

#### 4.2.3. The Analysis of the Detection Errors 

**Figure 10:**
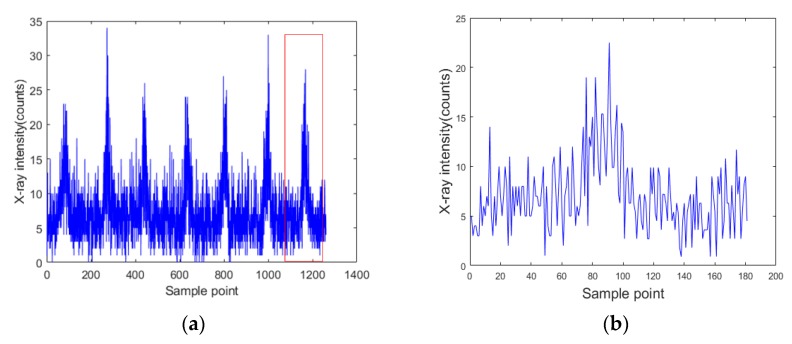

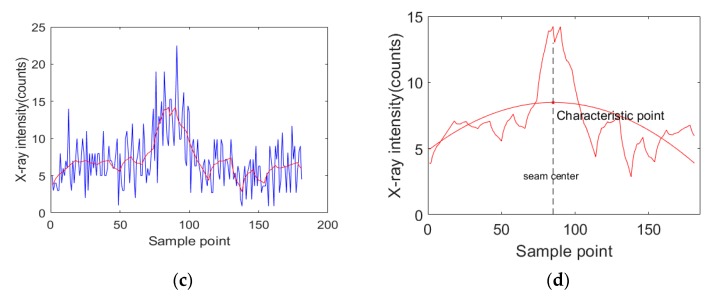
The whole procedure of X-ray intensity signal processing for single scanning period: (**a**) original signal; (**b**) read signals in single scanning period; (**c**) denoising; (**d**) curve fitting.

Table 1 shows the absolute error of fitting results for some processing methods. From Table 1, it can be seen that the absolute error of the processing algorithm based on wavelet packet was the smallest. At the same time, the correction before curve fitting was necessary, as can see that the absolute error dropped from 0.838 to 0.032 mm. However, this was only the detection error of a single experiment result. The reliability of the designed processing method was further verified by several experiments (Section 5). Compared with the average filter method and the Kalman filter method, the wavelet-packet-based algorithm obtained a better processing effect. Therefore, we chose the algorithm based on wavelet packet to process the acquired X-ray intensity signals. Figure 10 shows the whole signal processing procedure.

## 5. Detection Process Parameter Optimization

As described in Section 2, a well-collimated pencil-beam of X-rays was directed toward the specimen in the detection, and the X-rays backscattered from the specimen were collected by detector. In the above detection process, photon energy and X-ray intensity were gradually attenuated. The whole attenuation path of X-rays mainly includes the following sections: (1) the intensity attenuation of X-rays during the incident process; (2) the attenuation of intensity and energy of X-rays due to backscattering effect; (3) the intensity attenuation of the backscattered X-rays during their arrival from the backscattering position to the detector. In the attenuation path, the forms of interaction between X-ray and matter include: photoelectric effect, Compton scattering effect, pair production, Rayleigh scattering effect, Bremsstrahlung, ionization, and multiple scattering [26]. All or some of above interaction forms may take effect to cause the attenuation of X-ray energy and intensity along the attenuation path. All interactions work together during the first and third attenuation sections above. For the second attenuation section, the predominant form of interaction would be Compton scattering when relatively low-energy X-rays (10 keV–1 MeV) are used [16,27]. Therefore, a single Compton scattering model is suitable to approximate the physical process of X-ray and matter interaction during the above second attenuation section.

**Figure 11:**
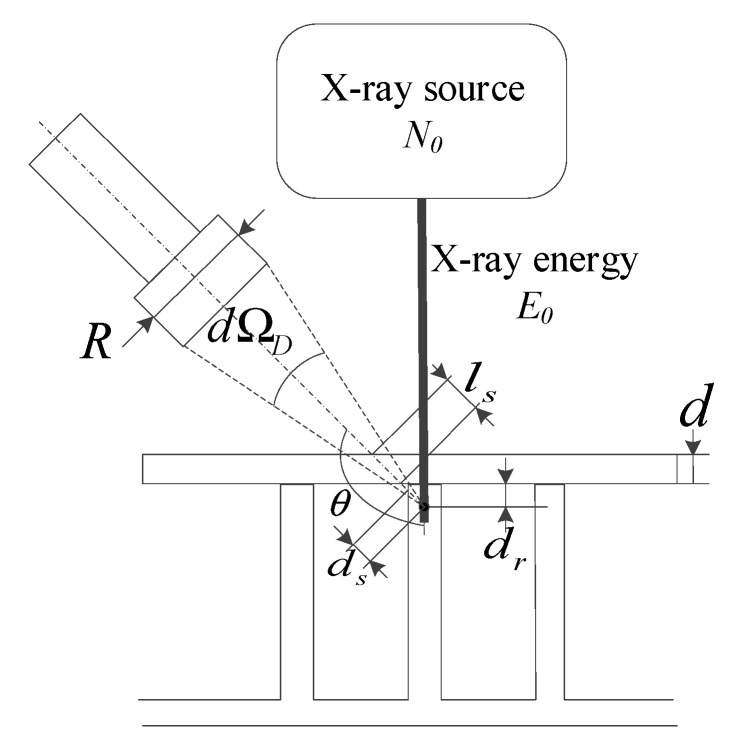
Modeling of X-ray backscattering detection.

According to the above analysis, an analytical model is put forward for calculating the intensity of backscattered X-rays acquired by the detector (Equation (11)). The influences of the three attenuation sections are included in the model, and the influences of other detection parameters (e.g., detector size and detection efficiency) on the acquired X-ray intensity are also considered. As shown in Figure 11, assuming that a single-energy X-ray beam with energy *E_0_* is irradiated on a section of the specimen, the X-ray intensity detected by the detector in unit time is:

(11) $$dN=N_{0}e^{-\mu_{0}l_{0}}n_{e}dvP\left(E_{0},\theta \right)e^{-\mu_{1}l_{1}}d\Omega_{D}\eta \;,$$

where *N* is the backscattered X-ray intensity detected by the detector per unit time; *N*_0_ is the intensity of the collimated incident X-ray beam (the number of X-ray photons passing through the unit area perpendicular to the direction of X-ray transmission in unit time); *n*_e_ is the average electron density in volume element irradiated by the incident X-ray beam; $P(E_{0},\theta )$ is the differential cross section for Compton scattering (describes the probability that an incident X-ray photon with energy *E_0_* is scattered into the unit solid angle of the direction scattering angle θ after interacting with an extra nuclear electron in the atom); $\mu_{0}$ is the linear attenuation coefficient for the incident X-ray beam; *l*_0_ is the attenuation length which the incident X-ray beam passes through the specimen; $\mu_{1}$ is the linear attenuation coefficient for the backscattered X-ray beam; *l*_1_ is the attenuation length which the backscattered X-ray pass through in the specimen; $d\Omega_{D}$ is the solid angle to the volume element irradiated by the incident X-ray beam; dv is the volume element irradiated by the incident X-ray beam; and η is the detection efficiency of the detector.

In Equation (11):

(12) $$l_{0}=d+d_{r},$$

where *d* is the attenuation length which the incident X-ray beam passes through in the face panel, *d_r_* is the attenuation length which the incident X-ray beam passes through in the core panel.

In Equation (11):

(13) $$l_{1}=l_{s}+d_{s},$$

where *l_s_* is the attenuation length which the backscattered X-ray pass through in the face panel, *d_s_* is the attenuation length which the backscattered X-ray pass through in the core panel.

In Equation (11):

(14) $$n_{e}=\frac{N_{a}Z\rho }{A},$$

where *N_a_* is the Avogadro constant, *Z* is the atomic number of the specimen material, ρ is the mass density of the specimen material, and *A* is the atomic mass number of the specimen material.

In Equation (11):

(15) $$P(E_{0},\theta )=\frac{d\sigma (\theta )}{d\Omega }=\left[\frac{r_{0}^{2}}{2\left[1+a_{0}{\left(1-\cos\theta \right)}^{2}\right]}\right]\left[1+\cos^{2}\theta +\frac{a_{0}^{2}{\left(1-\cos\theta \right)}^{2}}{1+a_{0}(1-\cos\theta )}\right],$$

where *r*_0_ is the classical electron radius, taking the value 2.818 × 10^−15^ m.

In Equation (15):

(16) $$a_{0}=\frac{E_{0}}{mc^{2}},$$

where $E_{0}$ is the energy of the incident X-ray, $mc^{2}$ is the rest mass energy of an electron, taking the value 0.511 MeV.

Equations (11)–(16) describe the relationship between the X-ray intensity acquired by the detector and the parameters of the detection system and specimen, such as the parameters of the X-ray source (i.e., energy, intensity), the relative position parameters between source, specimen, and detector (i.e., scattering angle, source–specimen distance, specimen–detector distance), and the material and structure parameters of the specimen (i.e., material density, material atomic number, thickness).

On the other hand, it is necessary to identify the difference in the X-ray intensities from positions with and without a core panel, and this difference needs to significantly exceed the noise fluctuation of the X-ray intensity signal at the position with a core panel.

Suppose the X-ray intensity detected from positions with and without core panels are *N_s_* and *N_b_*. So, the noise fluctuation *σ* of the X-ray intensity signal at the position with a core panel is *σ ≈ (N_s_)*^1/2^, and the difference in X-ray intensity from positions with and without core panel is ∆*N = N_s_ − N_b_*. Therefore, the condition for detecting weld should be ∆*N = K ×* σ, where *K* represents the signal-to-noise ratio of detection. Considering that the radiation dose is proportional to the power of the X-ray source (*E_0_ × N*_0_) in the detection process, in order to reduce radiation and improve the practicability of the detection method, the following optimal constraints under minimum radiation requirements can be obtained by taking *K* as 2:

(17) $$\forall .\;\min(E_{0}\times N_{0}),\;\Delta N\geq \;2\sigma$$

According to Equations (11)–(17), a range of detection system parameters were predetermined for a given specimen. The equations were utilized to obtain the appropriate parameters of the X-ray source in the detection system to reduce the radiation dose. To obtain the appropriate parameters of the X-ray source, the parameters of the specimen and the current detection system (listed in Table 2 and Table 3, respectively) were substituted into Equations (11)–(17). The backscattered X-ray intensities from positions with/without core panel (i.e., *N_s_* and *N_b_*) were calculated under different X-ray photon energies and X-ray beam intensities.

**Figure 12:**
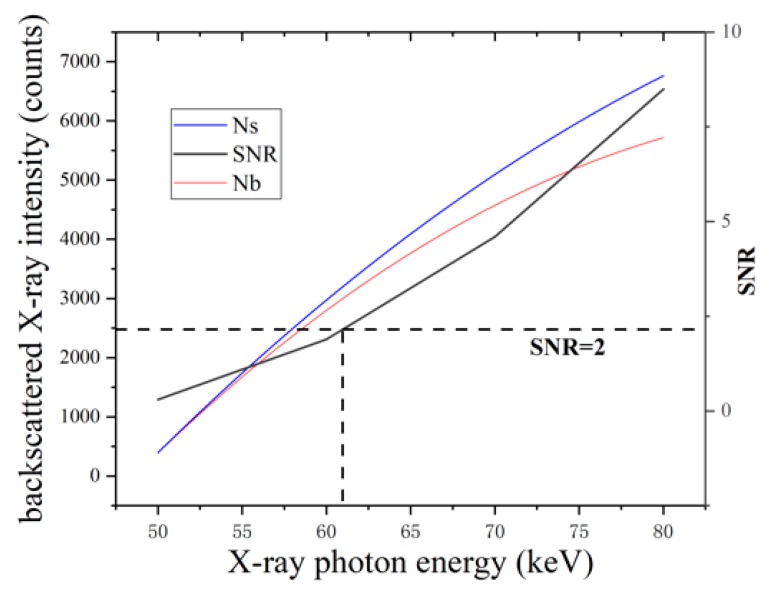
The effect of X-ray energy on detection. SNR: signal-to-noise ratio.

X-ray photon energy is a measurement of the penetrating power of the X-ray. According to the attenuation process described above, the poor penetrating power would render the failure of backscattered X-rays from the core panel incapable of reaching the detector, regardless of X-ray beam intensity, resulting in the failure of weld position detection. Accordingly, X-ray photon energy has a predominating effect on both the detection and radiation dose. Figure 12 shows the detected X-ray intensities (*N_s_* and *N_b_)* from positions with/without core panel under different photon energies while the X-ray beam intensity was constant. As shown in the figure, the X-ray photon energy should be at least 60–70 keV to achieve the required signal-to-noise ratio (SNR) under minimum radiation requirements.

On the other hand, the proposed detection method is essentially intended to identify the difference ∆*N* in the X-ray intensities from positions with/without core panel *N_s_*/*N_b_*, and ∆*N* needs to significantly exceed the noise fluctuation *σ* of the X-ray intensity signal at the position with the core panel. The difference ∆*N* is caused by the different amount of the X-rays which backscattered into the detector with or without the core panel. The intensity of these X-rays is not only determined by the core panel’s irradiated volume whose backscattered X-rays could reach the detector, but also the X-ray beam intensity which could reach the irradiated volume. 

The X-ray photon energy *E*_0_ determines the depth of irradiated volume in the core panel, while the beam diameter determines the intersection of the irradiated volume. Considering that the X-ray intensity which reaches the core panel was less than the intensity reaching the face panel due to the attenuation of specimen, and that the beam diameter ideally remains constant, the depth of irradiated volume in the core panel would make a greater increment effect than the reduction effect of lesser X-ray intensity reaching the irradiated volume on the detected intensity difference ∆*N* to achieve the SNR required for weld position detection.

**Figure 13:**
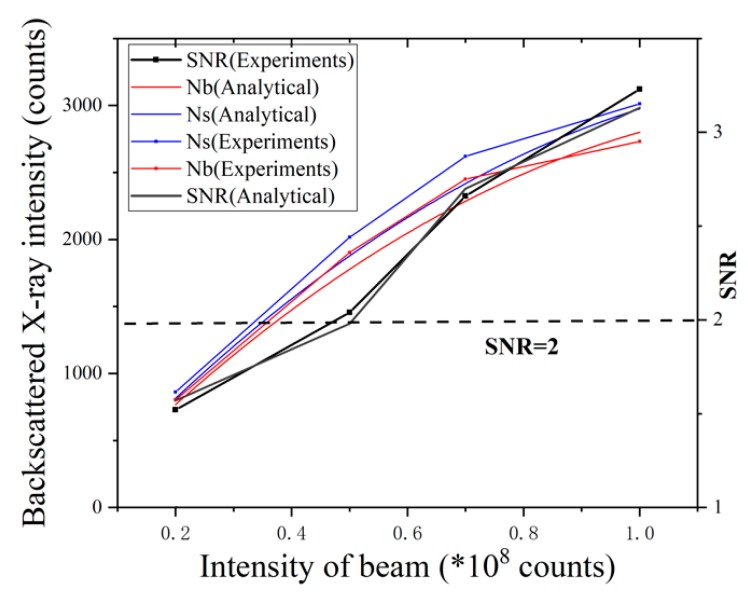
The effect of X-ray beam intensity on backscattered X-ray intensity signals.

As shown in Figure 13, with the X-ray beam intensity increased, the detected X-ray intensities (*N_s_* and *N_b_*) increased. The measurement noise *σ* and the difference of detected X-ray intensity with/without core panel ∆*N* also increased. However, due to the constant depth of irradiated volume determined by the X-ray photon energy, the reduction effect of less X-ray intensity reaching the irradiated volume gradually approached the increment effect of the depth of irradiated volume in the core panel on the detected intensity difference ∆*N* with increasing X-ray beam intensity. In contrast, the increment of noise *σ* kept increasing with the increment of X-ray beam intensity. This would cause a variable gradient of SNR of detection and influence the detection accuracy.

Considering the margin of detection parameters, the main detection parameters were determined as shown in Table 4. 

## 6. Weld Position Detection Experiments

### 6.1. Accuracy of Detection

**Figure 14:**
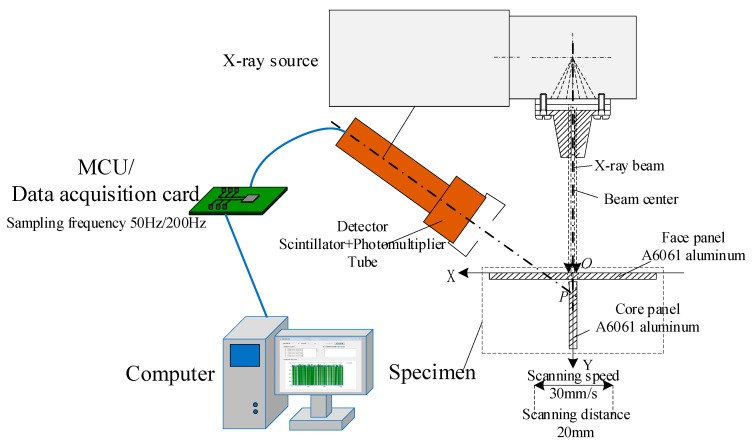
Schematic setup of the backscattered X-ray detection experiment.

**Figure 15:**
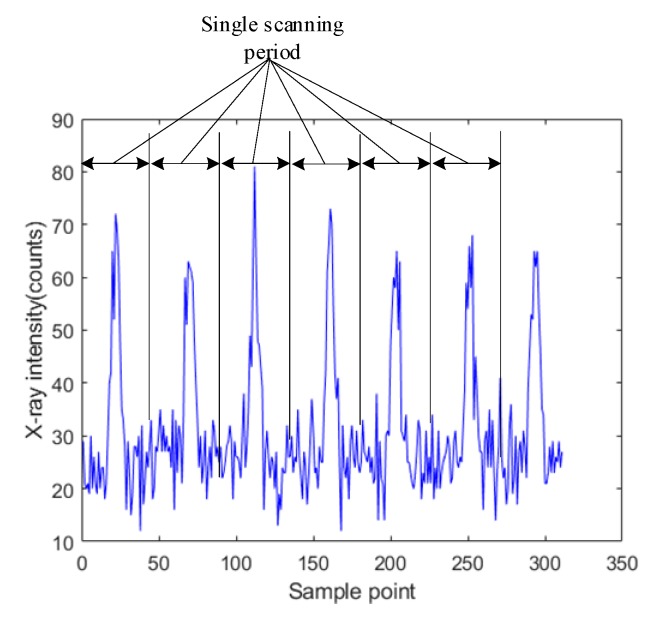
The original signals at 50 Hz sampling frequency.

To test the accuracy and reliability of the proposed signal processing method, repeated tests were carried out by using self-designed experimental equipment. Figure 14 shows the primary experimental parameters. The face panel and core panel were 3 mm thick. Figure 15 shows the original signals acquired in multiple scanning periods. By comparing the derived weld positions with the real seam center, the absolute errors of detection could be obtained.

**Figure 16:**
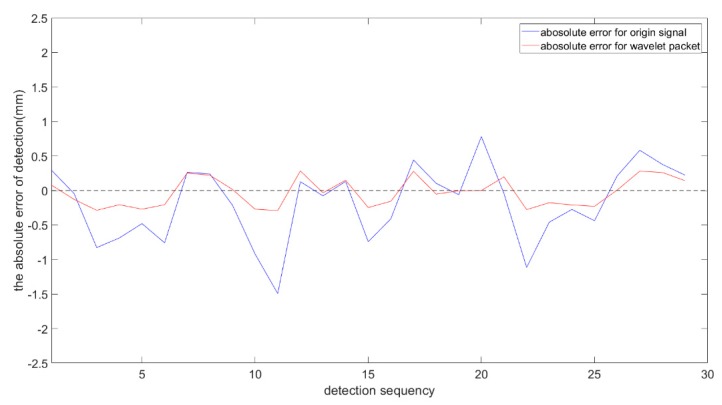
The absolute errors of detection in the experiments.

Figure 16 and Table 5 show the detection errors of the weld center from the original signal and from the processed signal based on wavelet packet, respectively. The blue curve indicates the absolute error of the detection before signal processing. The maximum absolute value of the errors was 1.527 mm. The red curve indicates the absolute error of the detection after signal processing. After the signal processing, the detection error was significantly reduced and the maximum absolute value was reduced to 0.287 mm, which is accurate enough to meet the detection requirements for a 3-mm-thick specimen.

### 6.2. Dynamic Performance of Detection

High welding speeds require high dynamic performance of the weld position detection system. With increased welding speed, higher sampling frequency is needed. To ensure a fixed interval between adjacent reference points (i.e., the seam-tracking accuracy), higher welding speed leads to a shorter scanning period. As the single scanning period decreases, a higher sampling frequency is required to maintain sufficient sample points in a single scanning period (i.e., sample accuracy). Therefore, increasing the sampling frequency can not only meet the requirements of higher welding speed but also a higher seam-tracking accuracy, so the influence of sampling frequency on the detection results is investigated in the paper.

As mentioned above, when the sampling frequency is increased, a shorter sampling period may reduce the number of X-ray photons collected in a single sampling period. The decreased X-ray intensity signal will lead to a larger relative standard error and smaller signal-to-noise ratio.

**Figure 17:**
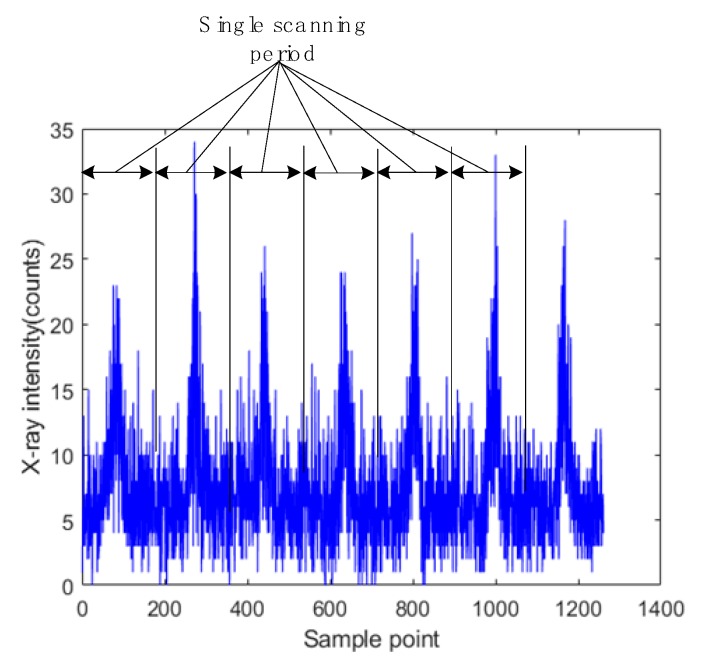
The X-ray intensity signal at 200 Hz sampling frequency.

Keeping the other experimental parameters and specimen fixed, the sampling frequency was increased from 50 to 200 Hz in the experiments. The acquired X-ray intensity signal is shown in Figure 17. It can be seen that the intensity of backscattered X-rays detected by the detector decreased significantly, the burr of the detection signal increased, and the signal-to-noise ratio of the detection signal decreased significantly.

**Figure 18:**
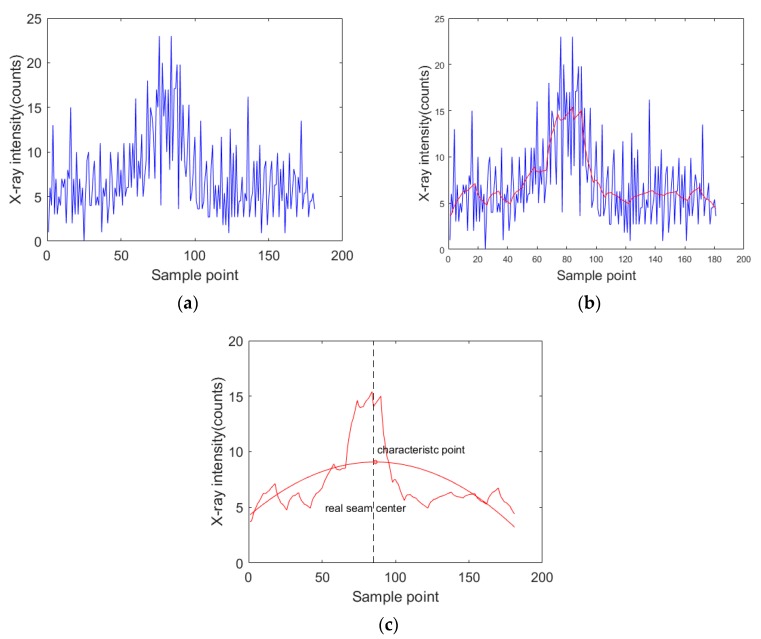
The processing procedure of X-ray intensity signal at a sampling frequency of 200 Hz: (**a**) read signals in a single scanning period; (**b**) denoising; (**c**) curve fitting.

As shown in Figure 18, the acquired signals were processed by the proposed processing method. Comparing the derived weld positions with the real seam center, the absolute errors of detection were obtained in several experiments.

**Figure 19:**
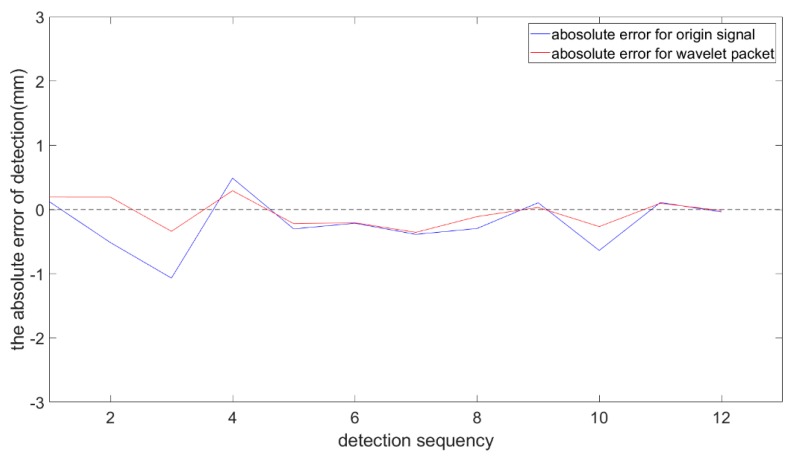
The absolute error of detection in the experiments.

Figure 19 and Table 6 show the detection errors of the weld center from the original signal and from the processed signal in several experiments. The blue curve indicates the detection error of the original signal. The maximum value of absolute errors was about 1.242 mm. The red curve indicates the absolute error of the detection after the signal processing. The detection error was significantly reduced. The absolute value was reduced to 0.340 mm, which is accurate enough to meet the requirements of weld position detection for specimens of 3 mm thickness. In terms of dynamic performance, the sampling frequency was 200 Hz. When the sample interval was 0.3 mm, the cross-section scanning of T-joint could be completed in 50 ms, which meets the requirement of general welding speed.

## 7. Conclusions

The paper investigates the weld position detection of T-joints from the face panel side of web-core sandwich panels. First, we designed an experimental system for the weld position detection of T-joints from the face panel side, which can quickly acquire the X-ray intensity signal backscattered by the specimen. Then, comparison of the processing results based on different processing algorithms indicated that the signal processing method based on wavelet packet was capable of reducing the noise and improving the detection accuracy significantly. Secondly, this paper establishes an analytical model to illustrate the relationship between backscattered X-ray intensity signal detected by the detector and the process parameters of the detection system and specimen. The proposed model was suitable for calculating and optimizing the detection process parameters required for detecting the weld position of a given specimen. Finally, several experiments were carried out on a 6061 aluminum alloy specimen with 3-mm-thick face panel and core panel. The characteristic values of the acquired X-ray intensity signals were extracted by the proposed processing method. From the aspect of detection accuracy, the maximum value of the detection errors was 0.340 mm, which is capable of meeting the detection accuracy requirement of 3-mm-thick core panels. From the aspect of dynamic performance, the minimum sampling period of the experiments was 5 ms. When the sample interval was 0.3 mm for a 3-mm-thick core panel, the cross section scanning of a T-joint could be completed in 50 ms, which is capable of meeting the requirement of general welding speed at the sampling frequency.

## Figures and Tables

**Table 1:** Comparison of detection error after signal processing.

Processing Algorithm	Absolute Error Before Correction (mm)	Absolute Error After Correction (mm)
Origin	0.916	0.105
Average filter	0.907	0.086
Kalman filter	1.029	0.214
Wavelet packet	0.838	0.032

**Table 2:** The primary specimen parameters.

Parameter Name	Parameter Value
Material type	AA6061 Aluminum
Thickness of cover panel (mm)	3
Thickness of core panel (mm)	3

**Table 3:** The primary process parameters of the detection system.

Parameter Name	Parameter Value
X-ray photon energy *E*_0_ (keV)	50/60/70/80
X-ray beam intensity *N*_0_ (counts)	2 × 10^7^/5 × 10^7^/7 × 10^7^/10^8^
Incident beam diameter (mm)	2
Diameter of the sensitive end of the detector (mm)	50
Scattering angle (°)	120
Distance between source and face panel (mm)	200

**Table 4:** The determined primary process parameters of X-ray backscatter detection system.

Parameter Name	Parameter Value
X-ray photon energy (keV)	60–70
X-ray beam intensity (counts)	10^8^
Incident beam diameter (mm)	2
Diameter of the sensitive end of the detector (mm)	50
Scattering angle (°)	120
The distance between source and face panel (mm)	200
The distance between detector and T-joint (mm)	120

**Table 5:** Comparison of detection errors at 50 Hz sampling frequency.

Processing Algorithm	Max Absolute Error (mm)	Max Error to Thickness of Core Panel	Standard Error (mm)	Standard Error to Thickness of Core	Standard Deviation (mm)
Origin signal	−1.527	50.9%	0.563	18.8%	0.542
Wavelet packet	−0.287	9.57%	0.205	6.83%	0.206

**Table 6:** Comparison of detection errors at 200 Hz sampling frequency.

Processing Algorithm	Max Absolute Error (mm)	Max Error to Thickness of Core Panel	Standard Error (mm)	Standard Error to Thickness of Core	Standard Deviation (mm)
Origin signal	−1.242	41.4%	0.453	15.1%	0.414
Wavelet packet	−0.340	11.3%	0.221	7.37	0.223
